# Current Real‐World Status of Off‐Label Under‐ and Over‐Dose of Direct Oral Anticoagulants After Atrial Fibrillation Ablation

**DOI:** 10.1111/jce.16560

**Published:** 2025-01-07

**Authors:** Tabito Kino, Akihiko Nogami, Kyoko Soejima, Kikuya Uno, Koichiro Kumagai, Takashi Kurita, Masayuki Fukuzawa, Atsushi Takita, Tomoko Ishizu, Kazutaka Aonuma, and other members of the RYOUMA Registry Investigators, Yuki Komatsu, Itsuro Morishima, Kenichi Hiroshima, Ritsushi Kato, Satoru Sakagami, Fumiharu Miura, Keisuke Okawa, Masahiko Gosho

**Affiliations:** ^1^ Department of Cardiology, Faculty of Medicine University of Tsukuba Tsukuba Japan; ^2^ Department of Cardiology Kyorin University School of Medicine Tokyo Japan; ^3^ Heart Rhythm Center, Tokyo Heart Rhythm Hospital Tokyo Japan; ^4^ Heart Rhythm Center, Fukuoka Sanno Hospital Fukuoka Japan; ^5^ Division of Cardiovascular Center Kindai University School of Medicine Osaka Japan; ^6^ Primary Medical Science Department Daiichi Sankyo Co. Ltd. Chuo‐ku Japan; ^7^ Data Intelligence Department Daiichi Sankyo Co. Ltd. Chuo‐ku Japan; ^8^ Department of Cardiology Ogaki Municipal Hospital Ogaki Japan; ^9^ Cardiovascular Division Kokura Memorial Hospital Fukuoka Japan; ^10^ Department of Arrhythmia Saitama Medical University International Medical Center Saitama Japan; ^11^ Department of Cardiology National Hospital Organization Kanazawa Medical Center Kanazawa Japan; ^12^ Department of Cardiovascular Medicine Hiroshima Prefectural Hospital Hiroshima Japan; ^13^ Department of Cardiovascular Medicine Kagawa Prefectural Central Hospital Takamatsu Japan; ^14^ Department of Biostatistics University of Tsukuba Tsukuba Japan

**Keywords:** atrial fibrillation, catheter ablation, direct oral anticoagulant therapy, off‐label overdose, off‐label underdose

## Abstract

**Background:**

Off‐label under‐ and overdosing of direct oral anticoagulants (DOACs) in patients with atrial fibrillation (AF) is not uncommon in real‐world practice.

**Objective:**

This study aimed to identify efficacy and safety of off‐label DOACs dose after AF ablation.

**Methods:**

The RYOUMA registry was a prospective multicenter study of Japanese patients who underwent AF ablation between 2017 and 2018. DOAC prescriptions were categorized into on‐label standard dose, on‐label reduced dose, off‐label underdose, and off‐label overdose.

**Results:**

The proportion of off‐label doses among patients after AF ablation varied depending on the type of DOAC, ranging from 13.5% to 34.9%. Of 2821 patients, 366 (13.0%) were prescribed an off‐label underdose and exhibited significantly higher CHADS_2_, CHA_2_DS_2_‐VASc, CHA_2_DS_2_‐VA, HELT‐E_2_S_2_, and HAS‐BLED scores, age, concomitant use of antiplatelets, and lower weight when compared to the on‐label standard dose (*n* = 1809). While the incidence of ischemic stroke after 1 year of off‐label underdose was notably low (0.28%), the rate of major bleeding was relatively high (1.7%). Off‐label overdose was prescribed to 134 patients (4.8%), who showed a significantly higher incidence of major bleeding (3.0%) compared to on‐label standard dose (0.91%; *p* = 0.02). The off‐label overdose group did not have any particular background and its thromboembolic risk was, conversely, low. The most likely cause of off‐label overdose was clinicians potentially overlooking dose criteria, including advanced age, low body weight, and low creatinine clearance.

**Conclusions:**

In patients after AF ablation, off‐label DOAC overdose was infrequent, but significantly associated with higher incidence of major bleeding during the remote period after AF ablation.

**Trial Registration:**

The study was registered as UMIN000026092 (University Hospital Medical Information Network‐Clinical Trial Registry).

## Graphical abstract

Real‐world ablation therapy with anticoagulants in management of atrial fibrillation for up to 1 year in Japan. DOAC, direct oral anticoagulant.

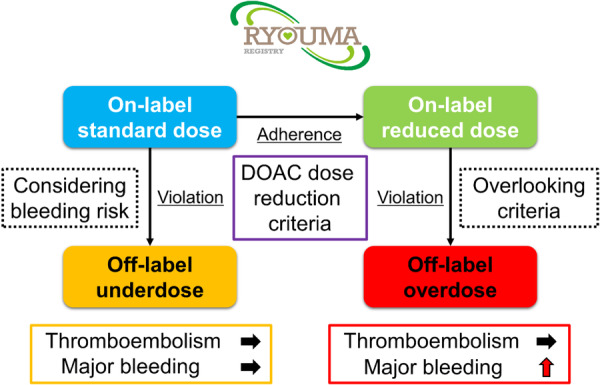

## 1 Introduction

Atrial fibrillation (AF) is the common cardiac arrhythmia worldwide [1]. Catheter ablation (CA) is currently the most effective treatment for AF [2, 3, 4], however, approximately 2%–3% of patients undergoing AF ablation develop periprocedural complications such as thromboembolism (0.2%) and major bleeding (0.9%) [5]. Optimal perioperative anticoagulation therapy is essential to reduce the risk of these complications, and an uninterrupted anticoagulant strategy including direct oral anticoagulants (DOACs) and warfarin is recommended before AF ablation [2, 3, 4, 6].

The Real‐world ablation therapY with anticOagUlants in Management of Atrial fibrillation (RYOUMA) registry was conducted to investigate the relationship between oral anticoagulants (OACs) therapy and efficacy/safety during the periprocedural and long‐term follow‐up periods in patients who underwent CA for nonvalvular AF in a real‐world setting [7]. The RYOUMA registry revealed that some patients received off‐label underdose (13.1%) and some received off‐label overdose (4.7%). “Off‐label” describes the otherwise proper use of prescription drugs at a dose that is not in accordance with the approved dose criteria. A real‐world database [8] and various meta‐analyses [9, 10, 11] have shown that off‐label underdosing of DOACs increases the risk of thromboembolism while not decreasing the risk of major bleeding, whereas off‐label overdosing of DOACs is associated with increased risks of thromboembolic and major bleeding events compared with on‐label doses. Since this evidence is not based on a CA setting, how off‐label under‐ or overdosing affects efficacy and safety profiles during and after AF ablation remains unclear. We, therefore, conducted a subanalysis of the RYOUMA registry focusing on off‐label under‐ and overdosing.

This study aimed to identify efficacy and safety outcomes during the periprocedural and long‐term follow‐up periods among patients who had undergone CA for nonvalvular AF, focusing on (1) on‐label standard dose versus off‐label underdose; and (2) on‐label reduced dose versus off‐label overdose. In addition, we aimed to identify reasons for off‐label overdose occurring counter to approved dose reduction criteria and the novel risk score for bleeding adjusted to DOACs in addition to the existing risk score.

## 2 Methods

### 2.1 Study Design

The design, methodology, and primary results of the RYOUMA registry have already been reported in detail [7]. In brief, the RYOUMA registry was a prospective multicenter observational study conducted from 2017 to 2018 in 62 institutions in Japan (*n* = 3170). All patients who underwent the scheduled first CA for nonvalvular AF were eligible for inclusion (*n* = 3072). In this subanalysis of the RYOUMA registry, patients using warfarin (*n* = 156) or not using OAC (*n* = 72) before CA were excluded. Demographic information from the patients was obtained during registration, and information was recorded during the periprocedural and remote phases.

### 2.2 Off‐ and On‐Label Dosing of DOACs

DOAC dose categories were defined according to the approved dose reduction criteria in Japan, as follows: (1) on‐label standard dose; (2) on‐label reduced dose; (3) off‐label underdose; and (4) off‐label overdose (Table 1). Unlike other DOACs, dabigatran does not have clear dose reduction criteria in Japan. Precautions for dosage were outlined in the package insert for patients at high risk of bleeding, such as creatinine clearance (CrCl) 30–50 mL/min, concomitant use of an oral P‐glycoprotein (P‐gp) inhibitor, age ≥ 70 years, and history of gastrointestinal (GI) bleeding. In this study, dose reduction criteria for dabigatran were defined based on these descriptions. We focused on on‐label standard dose versus off‐label underdose, and on‐label reduced dose versus off‐label overdose because patients' backgrounds may be similar. That is, “off‐label underdose,” where patients who should have received the standard dose were given a reduced dose (underdose), and “off‐label overdose,” where patients who should have received a reduced dose were given the standard dose (overdose).

**Table 1:** Dose reduction criteria for apixaban, dabigatran, edoxaban, and rivaroxaban.

	Apixaban	Dabigatran^a^	Edoxaban	Rivaroxaban
Dose reduction criteria according to the package insert in Japan (2017)	If two or more of the following criteria are met, the reduced dose should be used: a. Age ≥ 80 years b. Body weight ≤ 60 kg c. Serum creatinine ≥ 1.5 mg/dL	If any of the following criteria are met, the reduced dose should be considered: a. CrCl 30–50 mL/min b. Concomitant use of an oral P‐glycoprotein inhibitor c. Age ≥ 70 years d. History of GI bleeding	If any of the following criteria are met, the reduced dose should be used: a. Concomitant use of quinidine sulfate, verapamil hydrochloride, erythromycin, and cyclosporine b. CrCl 15–50 mL/min c. Body weight ≤ 60 kg	If the following criterion is met, the reduced dose should be used: a. CrCl 15–49 mL/min
On‐label standard dose	5 mg bid	150 mg bid	60 mg qd	15 mg qd
On‐label reduced dose	2.5 mg bid	110 mg bid	30 mg qd^b^	10 mg qd
Off‐label overdose	> 5 mg bid or > 2.5 mg bid despite meeting reduction criteria	> 150 mg bid or > 110 mg bid despite meeting reduction criteria	> 60 mg qd or > 30 mg qd despite meeting reduction criteria	> 15 mg qd or > 10 mg qd despite meeting reduction criterion
Off‐label underdose	< 5 mg bid despite not meeting reduction criteria or < 2.5 mg bid	< 150 mg bid despite not meeting reduction criteria or < 110 mg bid	< 60 mg qd despite not meeting reduction criteria or < 30 mg qd	< 15 mg qd despite not meeting reduction criterion or < 10 mg qd

Abbreviations: bid, twice daily; CrCl, creatinine clearance; GI, gastrointestinal; qd, once daily.

^a^ Since dabigatran does not have dose reduction criteria in Japan, both 150 mg bid and 110 mg bid were approved. Some precautions for dosage were only outlined in the package insert. Dose reduction criteria for dabigatran were defined based on these descriptions.

^b^ 15 mg qd was not approved in this research period.

### 2.3 Thromboembolic and Major Bleeding Risk Scores

Thromboembolic risk was evaluated using the CHADS_2_ score (congestive heart failure; hypertension; age ≥ 75 years; diabetes; previous stroke or transient ischemic attack [TIA] [doubled]) [9], the CHA_2_DS_2_‐VASc score (congestive heart failure; hypertension; age ≥ 75 years [doubled]; diabetes; previous stroke, TIA or thromboembolism [doubled]; vascular disease; age 65–74 years; and female sex) [10], the CHA_2_DS_2_‐VA score (congestive heart failure; hypertension; age ≥ 75 years [doubled]; diabetes; previous stroke, TIA or thromboembolism [doubled]; vascular disease; and age 65–74 years) [11], and the HELT‐E_2_S_2_ score (age 75–84 years, age ≥ 85 years [doubled], hypertension, low body mass index < 18.5 kg/m^2^, type of AF (persistent/permanent), and previous stroke [doubled]) [12]. Thromboembolic risk was considered high for CHADS_2_ score ≥ 2, CHA_2_DS_2_‐VASc score ≥ 3, CHA_2_DS_2_‐VA score ≥ 2, or HELT‐E_2_S_2 _≥ 2. Bleeding risk was also evaluated using the HAS‐BLED score (uncontrolled hypertension; renal dysfunction; liver dysfunction; prior stroke; previous bleeding; age ≥ 65 years; labile international normalized ratio; and use of aspirin or consumption of alcohol) [13]. Bleeding risk was considered high for HAS‐B(L)ED score ≥ 3. However, information on the element “L,” lability of the international normalized ratio, was not calculated because this factor is inappropriate for patients taking DOACs. We additionally analyzed the hazard ratio with the element of HAS‐BOED score including DOAC overdosing as “O” (off‐label overdosing) instead of “L.”

### 2.4 Clinical Outcomes

The primary endpoint was efficacy and safety properties to prevent major adverse events (ischemic stroke, systemic embolic events, and major bleeding events) during the 1‐year follow‐up period. Major bleeding events were defined according to International Society on Thrombosis and Hemostasis criteria [14], as evaluated by an event adjudication committee. Analyses were performed in two separate observation periods: the perioperative period (during and up to 30 days after CA); and the remote period (from 31 days up to 1 year after CA). Additionally, we evaluated the incidence of ischemic stroke or systemic embolic events in patients who discontinued DOAC therapy beyond 90 days postablation, as well as the incidence of major bleeding in those who maintained DOAC therapy.

### 2.5 Statistical Analyses

Continuous variables are expressed as median and interquartile range (IQRs). Categorical variables are summarized as the number and percentage. Baseline characteristics were compared using the *χ* ^2^ test for categorical variables and the Wilcoxon rank‐sum test for continuous variables. Kaplan–Meier methods were used to estimate adverse effects (AEs) during follow‐up. Cox regression hazard models were used to estimate hazard ratios and 95% confidence intervals (CIs) for AEs and the modified bleeding risk score during perioperative period and the remote period after CA. We considered a two‐sided *p* < 0.05 as statistically significant. Statistical analyses were performed using SAS version 9.4 (SAS Institute Inc., Cary, NC, USA).

## 3 Results

### 3.1 Patient Characteristics

**Figure 1:**
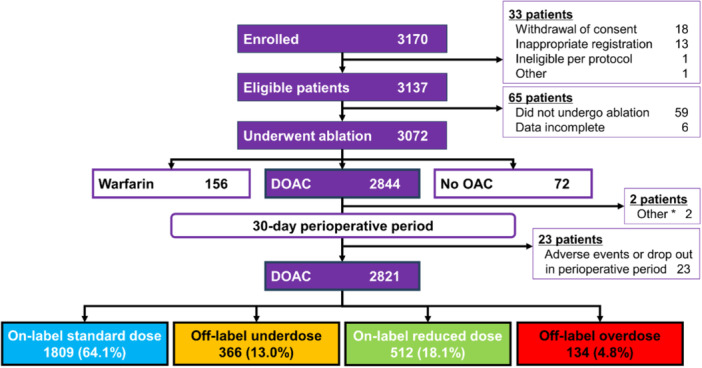
Flow diagram of enrolled patients. DOAC; direct oral anticoagulant, OAC; oral anticoagulant. * Patients who used a method of administration that violated the package insert.

A total of 2821 patients were categorized into four groups: on‐label standard dose (*n *= 1809), on‐label reduced dose (*n *= 512), off‐label underdose (*n *= 366), and off‐label overdose (*n *= 134) (Figure 1).

**Figure 2:**
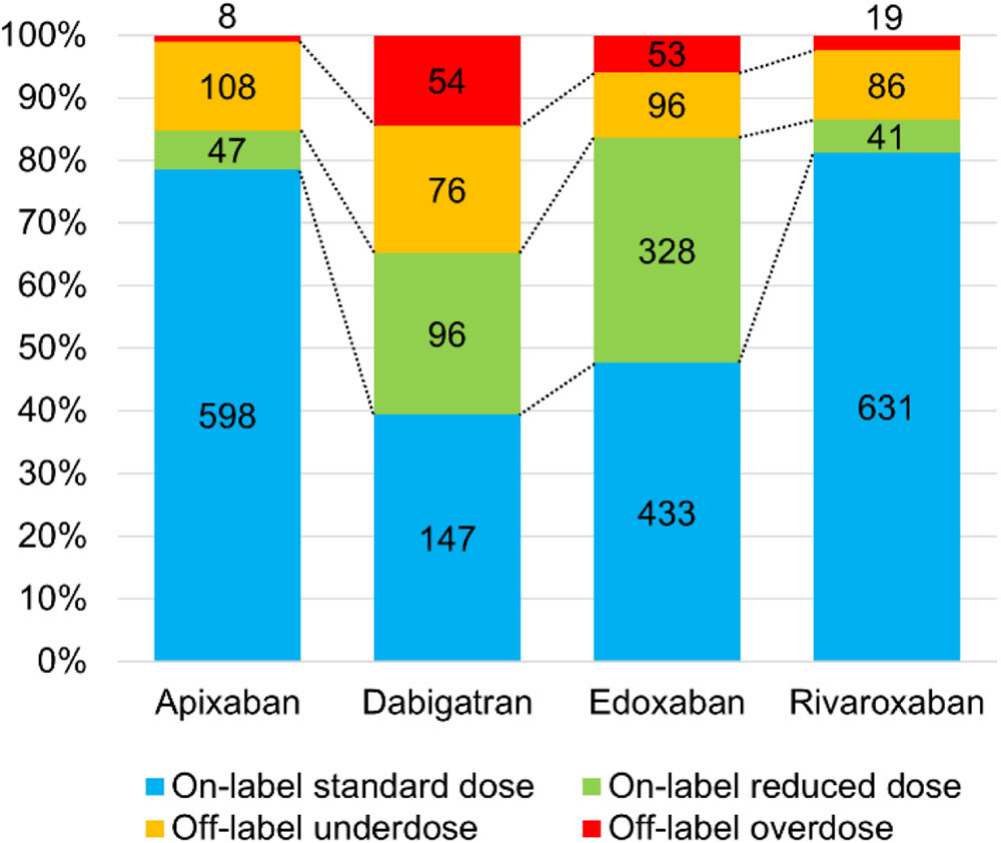
The proportions of dose categories for each DOAC. The numbers in the bars represent the number of patients. DOAC, direct oral anticoagulant.

**Figure 3:**
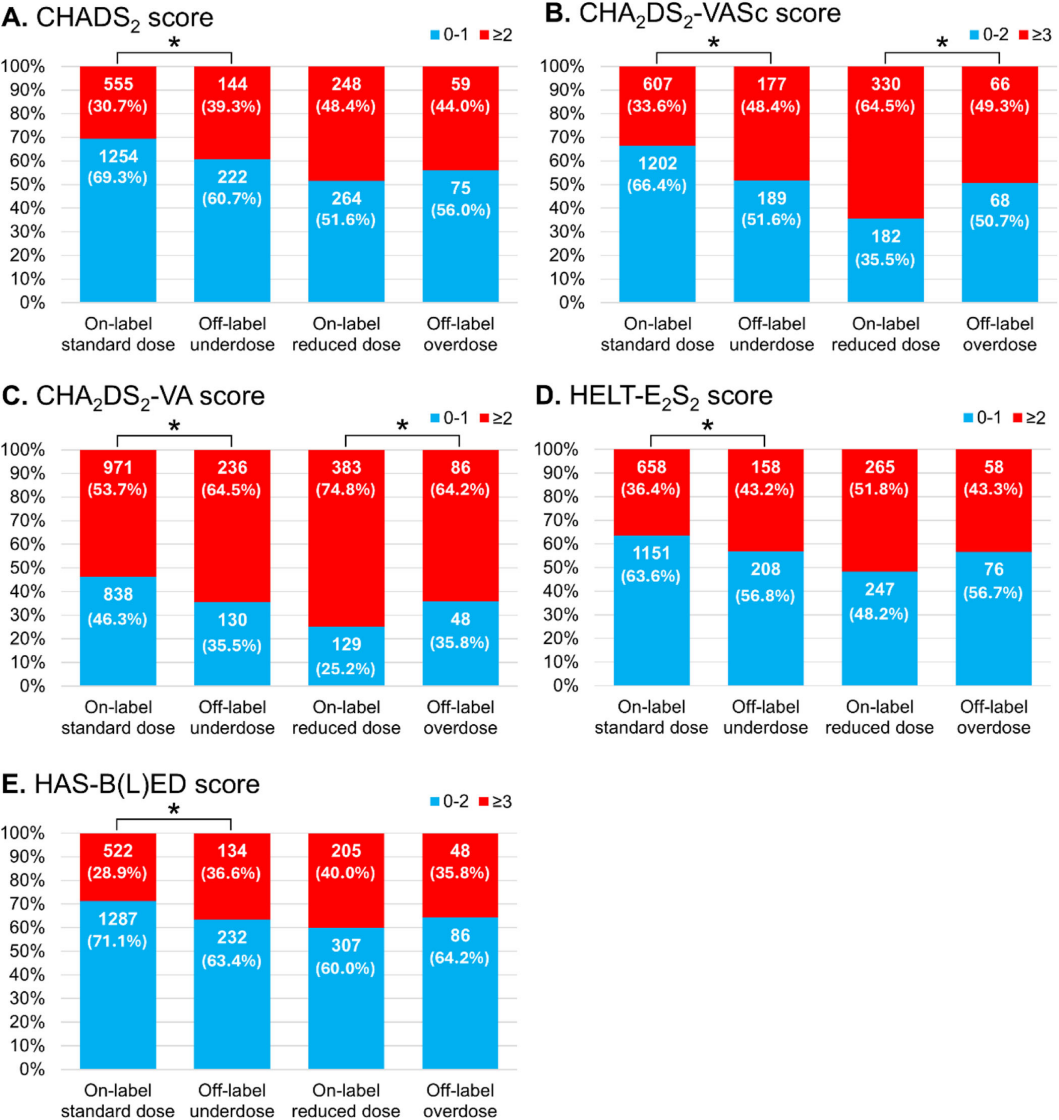
Stratified analyses according to the thromboembolic and bleeding risk scores. (A) CHADS_2_ score 0–1 or ≥ 2. (B) CHA_2_DS_2_‐VASc score 0–2 or ≥ 3. (C) CHA_2_DS_2_‐VA score 0–1 or ≥ 2. (D) HELT‐E_2_S_2_‐score 0–1 or ≥ 2. (E) HAS‐B(L)ED score 0–2 or ≥ 3. The numbers in the bars represent the number of patients and (%). * *p* < 0.05.

The proportions of patients categorized to each dose group for each DOACs are shown in Figure 2. The number of individuals taking each DOACs was as follows: 761 for apixaban, 373 for dabigatran, 910 for edoxaban, and 777 for rivaroxaban. Proportions of off‐label doses varied according to the type of DOAC, ranging from 13.5% (rivaroxaban) to 34.9% (dabigatran). Dabigatran showed the largest proportions of patients on off‐label underdose (20.4%) and overdose (14.5%). Furthermore, proportions of on‐label reduced dose were higher for dabigatran (25.7%) and edoxaban (36.0%). Patient characteristics are described in Tables 2 and 3. Compared to the on‐label standard dose group, patients in the off‐label underdose group were older (69.0 vs. 65.0 years, *p* < 0.001), included a higher proportion of females (32.2% vs. 20.8%, *p* < 0.001), and showed lower body weight (64.3 vs. 68.4 kg, *p* < 0.001) (Table 2). Thromboembolic and bleeding risk scores, CHADS_2_, CHA_2_DS_2_‐VASc, CHA_2_DS_2_‐VA, HELT‐E_2_S_2_, and HAS‐B(L)ED, were significantly higher in the off‐label underdose group compared to the on‐label standard dose group. Components of the HAS‐B(L)ED score, such as lower hemoglobin (13.9 g/dL vs. 14.4 g/dL, *p* < 0.001), abnormal renal function such as lower CrCl (70.7 vs. 84.1 mL/min, *p* < 0.001), and concomitant use of antiplatelets (14.2% vs. 7.0%, *p* < 0.001) were more frequent in the off‐label underdose group. Conversely, no significant differences in previous ischemic stroke, systemic embolism, hypertension, diabetes, or hepatic disorders were identified between on‐label standard dose and off‐label underdose groups. For stratified analyses, patients were divided into subgroups according to risk scores: CHADS_2_ score 0–1 or ≥ 2, CHA_2_DS_2_‐VASc score 0–2 or ≥ 3, CHA_2_DS_2_‐VA score 0–1 or ≥ 2, HELT‐E_2_S_2_ score 0–1 or ≥ 2, and HAS‐B(L)ED score 0–2 or ≥ 3 (Figure 3). The proportion of groups with high thromboembolic risk and high bleeding risk was significantly higher in the off‐label underdose group than in the on‐label standard dose group: CHADS_2_ score ≥ 2, 39.3% vs. 30.7%; CHA_2_DS_2_‐VASc score ≥ 3, 48.4% vs. 33.6%; CHA_2_DS_2_‐VA score ≥ 2, 63.5% vs. 53.7%; HELT‐E_2_S_2_ score ≥ 2, 43.2% vs. 36.4%; and HAS‐B(L)ED score ≥ 3, 36.6% vs. 28.9%, respectively (*p* < 0.05 each). The off‐label underdose group showed not only a higher risk of major bleeding, but also a higher risk of thromboembolism compared to the on‐label standard dose group.

**Table 2:** Baseline characteristics of enrolled patients between on‐label standard dose and off‐label underdose groups.

	**On‐label standard dose (*n*** = **1809)**	**Off‐label underdose (*n*** = **366)**	***p*** **value**
Age (years)	65.0 [58.0–70.0]	69.0 [61.0–75.0]	< 0.001
Sex, female	377 (20.8)	118 (32.2)	< 0.001
Body weight (kg)	68.4 [61.5–76.4]	64.3 [59.1–72.5]	< 0.001
BMI (kg/m^2^)	24.5 [22.4–26.9]	24.3 [22.2–26.6]	0.108
Never‐smoker	735 (40.6)	181 (49.5)	0.002
Nondrinker	641 (35.4)	169 (46.2)	< 0.001
Paroxysmal AF	1127 (62.3)	245 (66.9)	0.093
Long‐persistent AF	223 (12.3)	32 (8.7)	0.052
CHADS_2_ score	1.0 [0.0–2.0]	1.0 [0.0–2.0]	0.004
CHA_2_DS_2_‐VASc score	2.0 [1.0–3.0]	2.0 [1.0–4.0]	< 0.001
CHA_2_DS_2_‐VA score	2.0 [1.0–3.0]	2.0 [1.0–3.0]	< 0.001
HELT‐E_2_S_2_ score	1.0 [1.0–2.0]	1.0 [1.0–2.0]	0.003
HAS‐B(L)ED score	2.0 [1.0–3.0]	2.0 [1.0–3.0]	< 0.001
Antiarrhythmic drug	1286 (71.1)	291 (79.5)	< 0.001
Antiplatelet drug	127 (7.0)	52 (14.2)	< 0.001
Comorbidities			
Hypertension	1096 (60.6)	236 (64.5)	0.163
Diabetes	301 (16.6)	71 (19.4)	0.201
Heart disease	418 (23.1)	114 (31.1)	0.001
Kidney disease	115 (6.4)	42 (11.5)	< 0.001
Cerebrovascular disease	181 (10.0)	40 (10.9)	0.594
Thromboembolism	54 (3.0)	14 (3.8)	0.400
Hepatic disorder	118 (6.5)	17 (4.6)	0.174
Sick sinus syndrome	66 (3.6)	22 (6.0)	0.036
Other arrhythmias	257 (14.2)	70 (19.1)	0.016
Dementia	1 (0.1)	0 (0.0)	0.653
Cancer	155 (8.6)	32 (8.7)	0.913
GERD	306 (11.4)	37 (10.1)	0.479
Laboratory data			
Hct (%)	42.8 [40.1–45.4]	41.4 [38.8–44.1]	< 0.001
Hb (g/dL)	14.4 [13.4–15.4]	13.9 [12.9–14.9]	< 0.001
CrCl (mL/min)	84.1 [69.9–104.2]	70.7 [57.4–90.4]	< 0.001
HbA1c (%)	5.8 [5.5–6.1]	5.8 [5.5–6.3]	0.125
PT‐INR	1.2 [1.1–1.3]	1.2 [1.1–1.3]	0.096
APTT (sec)	31.9 [29.8–35.0]	32.3 [29.9–35.6]	0.324

*Note:* Data are shown as median [IQR, Q1–Q3] or *n* (%). Categorical variables were tested using the *χ* ^2^ test, while continuous variables were assessed using Wilcoxon's rank‐sum test.

Abbreviations: AF, atrial fibrillation; APTT, activated partial thromboplastin time; BMI, body mass index; CrCl, creatinine clearance; GERD, gastroesophageal reflux disease; Hb, hemoglobin; HbA1c, hemoglobin A1c; Hct, hematocrit; PT‐INR, prothrombin time‐international normalized ratio; SSS, sick sinus syndrome.

Compared to the on‐label reduced‐dose group, patients in the off‐label overdose group were younger (71.0 vs. 74.0 years, *p* < 0.001), with a smaller proportion of females (30.6% vs. 55.5%, *p* < 0.001), and higher body weight (60.0 vs. 54.0 kg, *p* < 0.001) (Table 3). While other risk scores were similar, CHA_2_DS_2_‐VASc was significantly lower in the off‐label overdose group compared to the on‐label reduced‐dose group. In addition, CrCl (71.7 vs. 57.1 mL/min, *p* < 0.001) and hemoglobin (14.1 vs. 13.3 g/dL, *p* < 0.001) were higher in the off‐label overdose group. The proportion of patients with high CHA_2_DS_2_‐VASc and CHA_2_DS_2_‐VA score was significantly lower in the off‐label overdose group compared with the on‐label reduced‐dose group (49.3% vs. 64.5%; and 64.2% vs. 74.8%, respectively [*p* < 0.05 each]) in stratified risk score analyses (Figure 3B and 3C). The off‐label overdose group was considered to show a lower risk of thromboembolism and similar risk of major bleeding events compared to the on‐label reduced‐dose group.

**Table 3:** Baseline characteristics of enrolled patients between on‐label reduced‐dose and off‐label overdose groups.

	**On‐label reduced dose (*n*** = **512)**	**Off‐label overdose (*n*** = **134)**	***p*** **value**
Age (years)	74.0 [69.0–78.5]	71.0 [63.0–75.0]	< 0.001
Sex, female	284 (55.5)	41 (30.6)	< 0.001
Body weight (kg)	54.0 [49.5–58.8]	60.0 [56.0–70.0]	< 0.001
BMI (kg/m^2^)	21.7 [19.8–23.7]	22.9 [21.1–25.2]	< 0.001
Never‐smoker	321 (62.7)	55 (41.0)	< 0.001
Nondrinker	297 (58.0)	60 (44.8)	0.006
Paroxysmal AF	354 (69.1)	79 (59.0)	0.026
Long‐persistent AF	38 (7.4)	16 (11.9)	0.092
CHADS_2_ score	1.0 [0.0–2.0]	1.0 [0.0–2.0]	0.127
CHA_2_DS_2_‐VASc score	3.0 [2.0–4.0]	2.0 [1.0–4.0]	< 0.001
CHA_2_DS_2_‐VA score	2.0 [1.0–3.0]	2.0 [1.0–3.0]	0.028
HELT‐E_2_S_2_ score	2.0 [1.0–2.0]	1.0 [1.0–2.0]	0.052
HAS‐B(L)ED score	2.0 [1.0–3.0]	2.0 [1.0–3.0]	0.150
Antiarrhythmic drug	386 (75.4)	84 (62.7)	0.003
Antiplatelet drug	57 (11.1)	9 (6.7)	0.133
Comorbidities			
Hypertension	306 (59.8)	71 (53.0)	0.156
Diabetes	80 (15.6)	25 (18.7)	0.397
Heart disease	170 (33.2)	45 (33.6)	0.934
Kidney disease	63 (12.3)	8 (6.0)	0.037
Cerebrovascular disease	74 (14.5)	16 (11.9)	0.455
Thromboembolism	28 (5.5)	2 (1.5)	0.052
Hepatic disorder	33 (6.4)	7 (5.2)	0.601
SSS	39 (7.6)	7 (5.2)	0.338
Other arrhythmias	96 (18.8)	19 (14.2)	0.016
Dementia	13 (2.5)	1 (0.7)	0.204
Cancer	87 (17.0)	12 (9.0)	0.021
GERD	70 (13.7)	33 (24.6)	0.002
Laboratory data			
Hct (%)	39.8 [36.7–42.2]	42.3 [38.7–44.7]	< 0.001
Hb (g/dL)	13.3 [12.2–14.2]	14.1 [12.9–14.8]	< 0.001
CrCl (mL/min)	57.1 [46.2–71.2]	71.7 [48.5–85.9]	< 0.001
HbA1c (%)	5.8 [5.5–6.1]	5.8 [5.5–6.2]	0.276
PT‐INR	1.2 [1.1–1.3]	1.2 [1.1–1.2]	0.054
APTT (sec)	31.9 [29.1–35.5]	34.1 [30.5–39.2]	< 0.001

*Note:* Data are shown as median [IQR, Q1–Q3] or *n* (%). Categorical variables were tested using the *χ* ^2^ test, while continuous variables were assessed using Wilcoxon's rank‐sum test.

Abbreviations: AF, atrial fibrillation; APTT, activated partial thromboplastin time; BMI, body mass index; CrCl, creatinine clearance; GERD, gastroesophageal reflux disease; Hb, hemoglobin; HbA1c, hemoglobin A1c; Hct, hematocrit; PT‐INR, prothrombin time‐international normalized ratio; SSS, sick sinus syndrome.

### 3.2 Efficacy and Safety Outcomes

**Figure 4:**
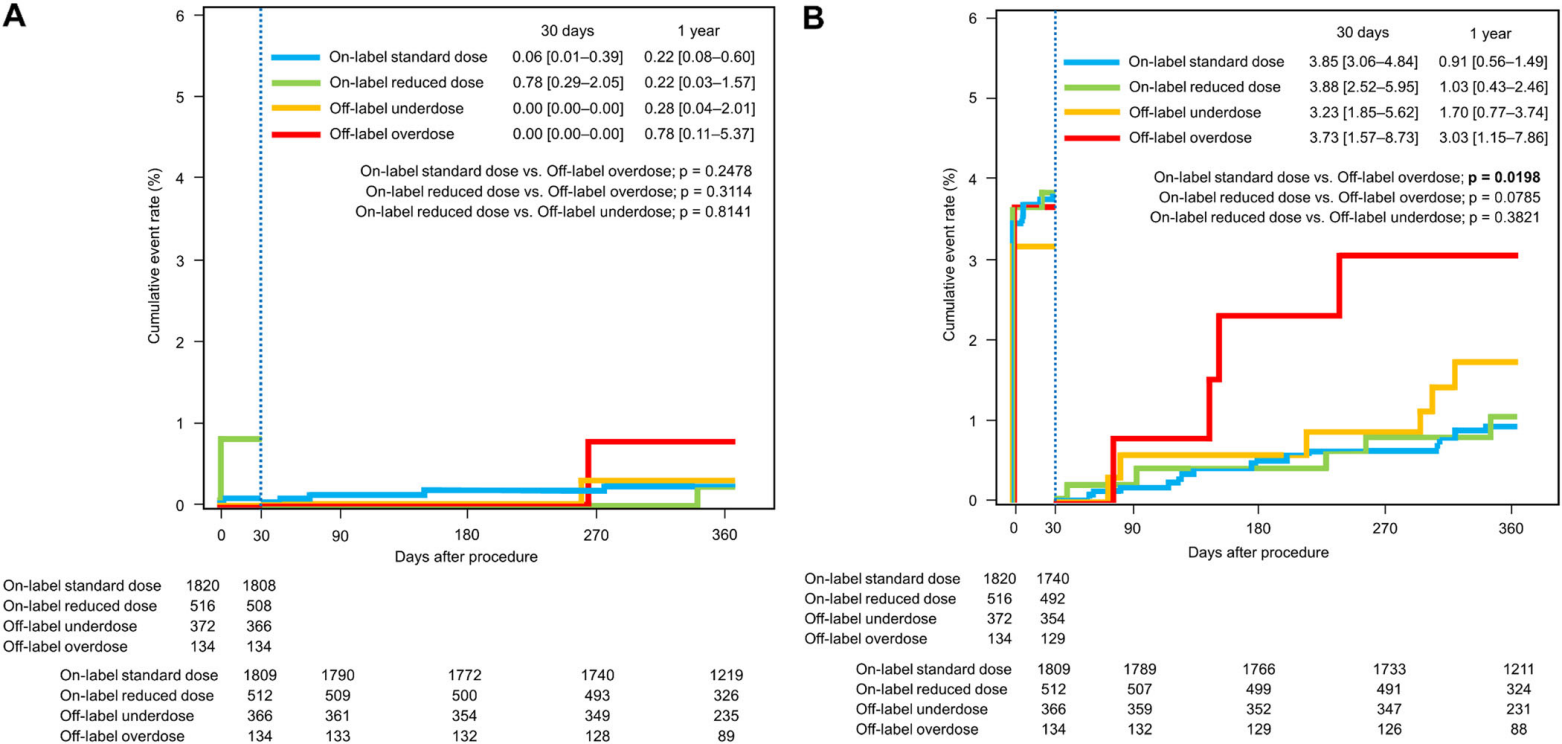
Kaplan–Meier plot of the time to the first major adverse events adjudicated. The incidence of major adverse events (ischemic stroke/systemic embolic events or major bleeding events) were separately analyzed during the perioperative period (during and up to 30 days after catheter ablation) and during the remote period (from 31 days and up to 1 year after catheter ablation). (A) The cumulative event rates of ischemic stroke/systemic embolic events. Thromboembolic event rates were similarly low in each categorized dose group during both the perioperative and remote periods. (B) The cumulative event rates of major bleeding. Major bleeding event rates during the periprocedural period were high in all categorized dose groups. The major bleeding event rate at 1 year in off‐label overdose group was significantly higher compared with on‐label standard dose group.

Cumulative event rates for efficacy and safety outcomes were analyzed separately during the periprocedural period (during and up to 30 days after CA) and remote period (from 31 days up to 1 year after CA). Thromboembolic event rates for each categorized dose group at 30 days after CA were similarly low, ranging from 0.00% to 0.78%, (Figure 4A). During the remote period after CA, thromboembolic event rates for each categorized dose group at 1 year after CA were similarly low, ranging from 0.22% to 0.78%. No significant differences in thromboembolic event rates during the periprocedural and remote periods were evident among the categorized dose groups. Major bleeding event rates during the periprocedural period were high in all categorized dose groups, ranging from 3.23% to 3.88% (Figure 4B). No significant differences in rates of major bleeding events were seen during the periprocedural period among categorized dose groups. During the remote period after CA, the major bleeding event rate at 1 year was significantly higher in the off‐label overdose group (3.03%, 95% CI 1.15–7.86) compared with the on‐label standard dose group (0.91%, 95% CI 0.43–1.49; *p* = 0.020). Although no significant difference was apparent, the major bleeding event rate at 1 year was higher in the off‐label underdose group (1.70%, 95%CI 0.77–3.74) compared with on‐label dose groups.

### 3.3 Deviations From Dose Reduction Criteria in the Off‐Label Overdose Group

**Figure 5:**
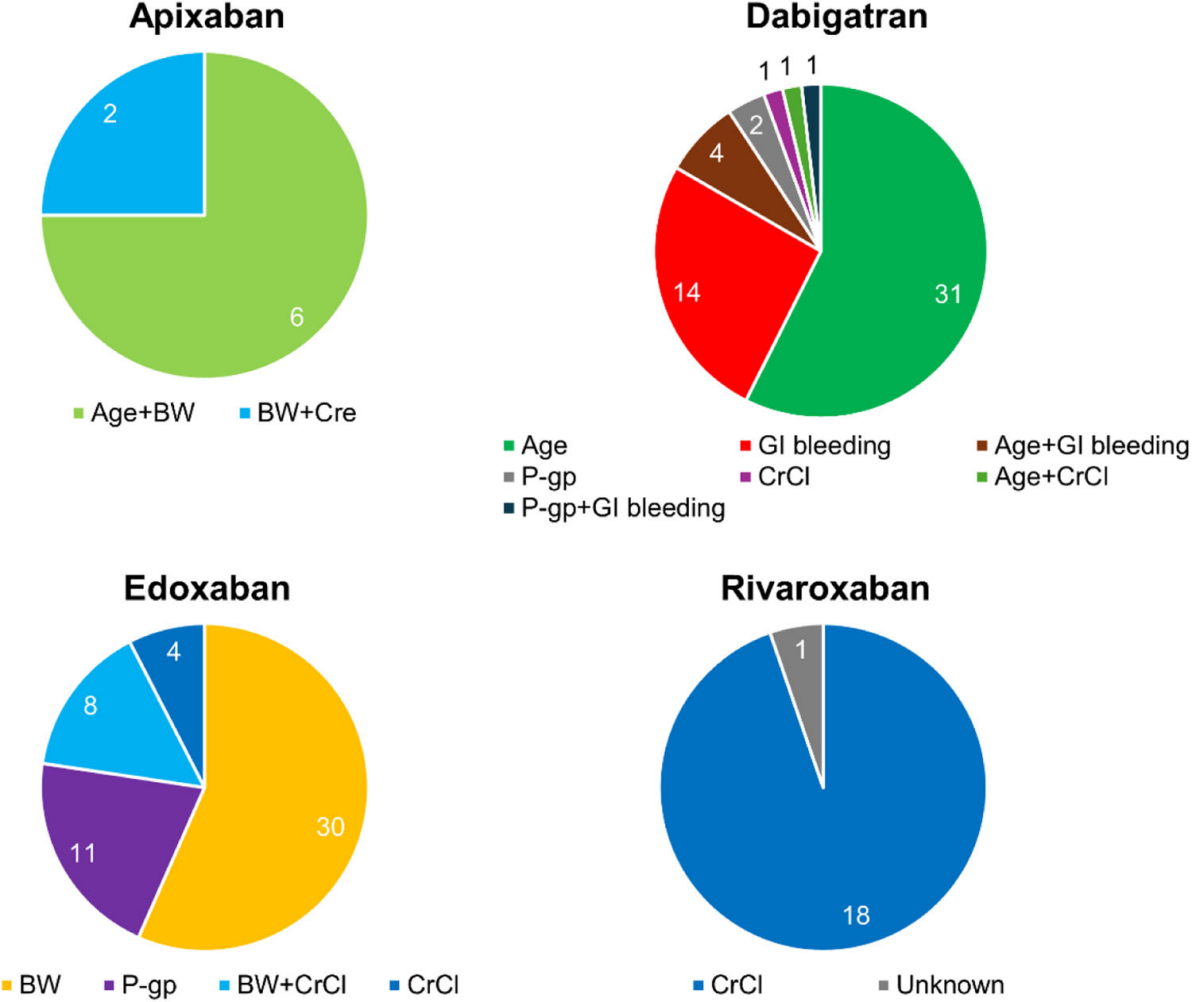
Missing dose reduction criteria in off‐label overdose for each DOAC. The numbers represent the number of patients. BW, body weight; CrCl, creatinine clearance; Cre, creatinine; GI, gastrointestinal; P‐gp, p‐glycoprotein.

Figure 5 shows the dose reduction criteria that were deviated from in the off‐label overdose group. With apixaban, low body weight was a criterion that was commonly deviated from. For dabigatran, the deviated criteria were complex, but elderly status and GI bleeding were the most frequent (90.7%). With edoxaban, the majority of deviations involved low body weight (71.7%), with concomitant use of a P‐gp inhibitor or low CrCl as the next most common reasons. For rivaroxaban, low CrCl was the only criterion deviated from.

### 3.4 Novel Risk Factors for Major Bleeding in Patients Taking DOACs

Since information on the element “L,” lability of the international normalized ratio, was inappropriate for patients taking DOACs, we analyzed the hazard ratio using the element of HAS‐BOED score including DOAC overdosing as “O.” In patients who underwent CA for AF and were receiving DOACs, previous bleeding (hazard ratio 2.56, 95% CI 1.05–6.24), DOAC overdose (hazard ratio 2.99, 95% CI 1.05–8.55), and age > 75 years (hazard ratio 4.06, 95% CI 1.66–9.96) were significantly associated with major bleeding (Table 4).

**Table 4:** Multiple Cox regression model for major bleeding.

Variable			Patients (*n*)	Major bleeding events (*n*)	Hazard ratio	95% CI
H	Hypertension	Yes	1709	22 (1.3)	1.60	0.74–3.47
A	Abnormal renal or liver function	Yes	416	3 (0.7)	0.62	0.19–2.02
S	Stroke	Yes	232	3 (1.3)	1.21	0.37–3.99
B	Bleeding	Yes	243	6 (2.5)	2.56*	1.05–6.24
O	DOAC overdose	Yes	134	4 (3.0)	2.99*	1.05–8.55
E	Elderly	< 65	1070	7 (0.7)	Ref.	
		65–75	1185	9 (0.8)	1.15	0.43–3.09
		> 75	566	15 (2.7)	4.06*	1.66–9.96
D	Drug or alcohol	Yes	1352	18 (1.3)	1.49	0.73–3.04

*Note:* Data are shown as *n* (%). Hazard ratio was estimated.

Abbreviations: 95% CI, 95% confidence interval; DOAC, direct oral anticoagulant; Ref., reference.

* *p* < 0.05.

### 3.5 The Thromboembolic Outcomes in Patients Who Discontinued DOACs and the Major Bleeding Outcomes in Those Who Continued DOACs 90 Days After AF Ablation

**Figure 6:**
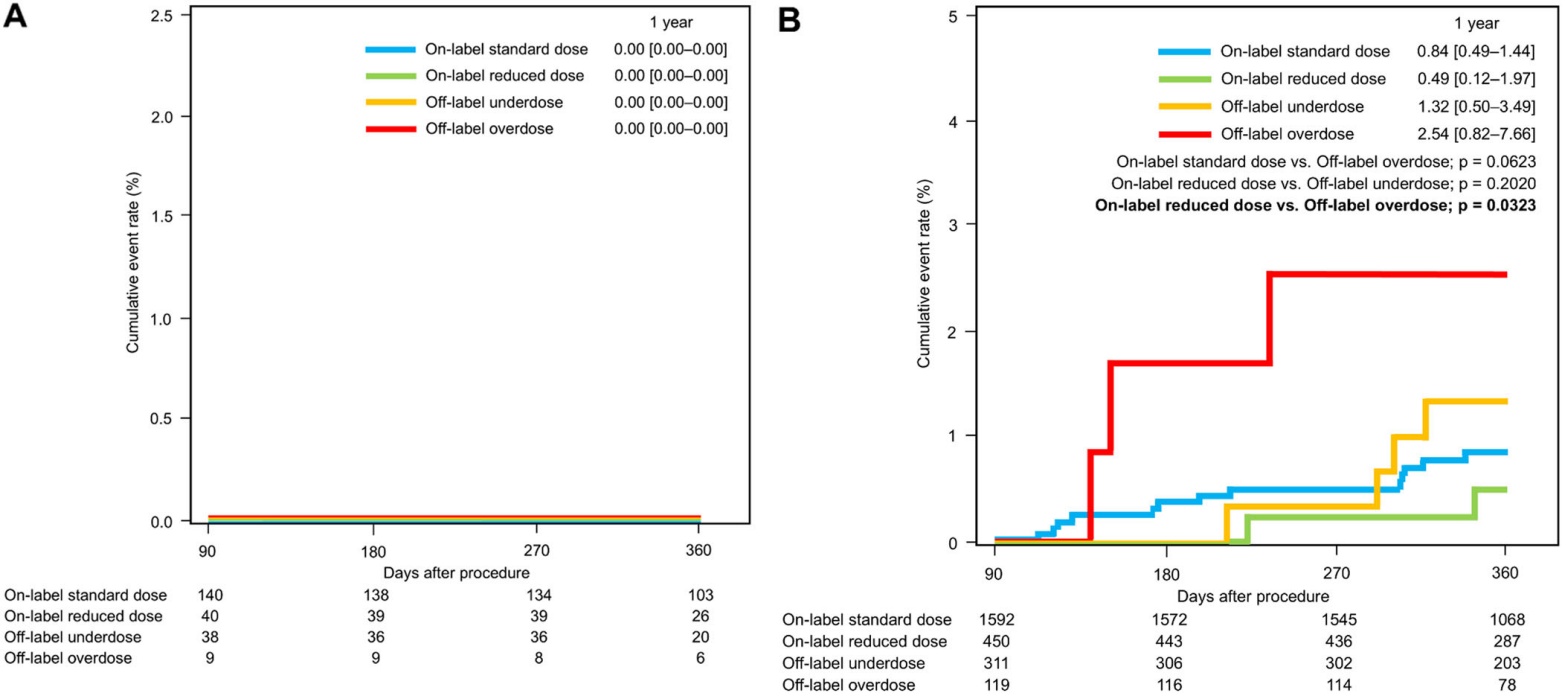
Kaplan–Meier plot of the time to the first major adverse events adjudicated. The incidence of major adverse events (ischemic stroke/systemic embolic events or major bleeding events) were separately analyzed during the late period (from 90 days and up to 1 year after catheter ablation). (A) The cumulative event rates of ischemic stroke/systemic embolic events in patients who discontinued DOACs beyond 90 days after AF ablation. No thromboembolic events were observed in each categorized dose group. (B) The cumulative event rates of major bleeding in patients who continued DOACs. The major bleeding event rate at 1 year in off‐label overdose group was significantly higher compared with on‐label reduced‐dose group.

A total of 327 patients discontinued DOAC therapy beyond 90 days after undergoing AF ablation, with follow‐up extending up to 1 year. No cases of stroke or systemic embolism were reported in any of the four groups in Figure 6A. Conversely, 2472 patients continued DOAC therapy after AF ablation until either the date of major bleeding event or the end of follow‐up period. The 1‐year major bleeding event rate was significantly higher in the off‐label overdose group (2.54%, 95% CI 0.82–7.66) compared to the on‐label reduced‐dose group (0.49%, 95% CI 0.12–1.97; *p* = 0.032). Although not statistically significant, the 1‐year major bleeding event rate was also higher in the off‐label underdose group (1.32%, 95% CI 0.50–3.49) compared to the on‐label dose groups (Figure 6B).

## 4 Discussion

### 4.1 Major Findings

The present study revealed the prevalence of off‐label DOAC prescription among patients registered to the RYOUMA registry following CA of AF, as well as the safety outcomes of off‐label under‐ and overdosing. The proportion of off‐label doses among patients after AF ablation varied depending on the type of DOAC, ranging from 13.5% with rivaroxaban to 34.9% with dabigatran. Off‐label underdosing was observed in 366 patients (13.0%), with a notably low incidence rate of ischemic stroke at 1 year (0.28%), but a relatively high rate of major bleeding (1.7%). Off‐label overdosing was prescribed to 134 patients (4.8%), and the incidence of major bleeding was significantly higher (3.0%) compared to the on‐label standard dose group (0.91%, *p* = 0.02). The most likely cause of off‐label overdosing was clinicians potentially overlooking dose criteria such as advanced age, low body weight, and low CrCl.

### 4.2 Off‐Label Dosing of DOACs

Thromboembolism is the most important complication in patients with AF, necessitating appropriate administration of OACs. Some previous meta‐analyses have reported that off‐label underdose is associated with increased risks of stroke or systemic embolism [15, 16], but other studies have not found such associations [8, 17, 18, 19]. On the other hand, off‐label overdosing reveals an increase in not only major bleeding, but also thromboembolic events [15, 16, 17]. However, the majority of previous studies have predominantly focused on non‐AF ablation patients. Since AF is likely to be suppressed after ablation, research targeting patients after AF ablation is important.

Wakamatsu et al. implemented a multicenter registry after CA for AF [20] and reported annualized thromboembolic and major bleeding event rates of 0.47% and 0.70% in the off‐label underdose group, compared to 0.74% and 0.73%, respectively, in the appropriate standard‐dose group. In the present study, rates of thromboembolic and major bleeding events at 1 year in the off‐label underdose group were 0.28% and 1.7%, compared to 0.22% and 0.91%, respectively, in the on‐label standard dose group. Thromboembolic events were thus extremely uncommon in all categorized dose groups, which may be attributed to the high level of AF suppression following CA. However, the reasons for the slightly higher rate of major bleeding events in the off‐label underdose group in our study remain unclear (Figure 3). When comparing background characteristics between the on‐label standard dose and off‐label underdose groups, the off‐label underdose group showed higher risks of both thromboembolism and major bleeding (Table 2).

Previous studies have summarized the drivers leading to off‐label underdosing as a means of avoiding bleeding complications as follows: older age, history of minor bleeding events, hypertension, congestive heart failure, concomitant use of drugs predisposing to bleeding, and low CrCl [21, 22, 23]. Some factors were in common with the present study. Clinicians may have prioritized the risk of major bleeding events in patients after CA of AF, assuming a lower risk of thromboembolism. Indeed, prescription of a standard dose to this group might well have led to a greater frequency of major bleeding events.

### 4.3 Off‐Label Overdosing

In a large cohort study conducted in the United States, off‐label overdosing of DOACs was prescribed more frequently in patients with worse renal function [23]. In our study, many dose reduction criteria were deviated from in the off‐label overdose group. The most common criteria were advanced age, low body weight, and low CrCl. Among DOACs, the proportion of off‐label overdosing was highest for dabigatran (14.5%). Advanced age and GI bleeding were common deviated criteria with dabigatran. However, dabigatran lacks clear dose reduction criteria in Japan. Some precautions for dosage were only outlined in the package insert, and dose reduction criteria for dabigatran in this study were defined based on those descriptions. The fact that the dosage of dabigatran is left to the discretion of the clinician is speculated to lead to an increase in “off‐label” overdosing. On the other hand, low body weight was a commonly deviated criterion for apixaban and edoxaban, whereas low CrCl was the only reduction criterion for rivaroxaban. While cutoff values for body weight and CrCl were consistent across DOACs where described, age cutoffs differed between apixaban (> 80 years) and dabigatran (> 70 years). This may also have contributed to the increased likelihood of “off‐label” overdosing with dabigatran. Since age, body weight, and CrCl can also change over time, clinicians must remain vigilant regarding the current status of patients.

### 4.4 Novel Risk Factors for Bleeding in Patients Taking DOACs

The HAS‐BLED score was developed to assess the 1‐year risk of major bleeding in patients with AF receiving therapeutic anticoagulation. However, this score was created for patients treated with vitamin K antagonists before the introduction of DOACs. Since our study found that off‐label overdosing with DOACs represents a risk factor for major bleeding, we analyzed the hazard ratios of a new risk co‐factor “O,” off‐label overdose, in addition to the other components of the HAS‐B(L)ED score (Table 4) and identified that off‐label DOAC overdose was significantly associated with major bleeding. Recently, new “DOAC score” has been proposed and shown to have stronger performance than the HAS‐BLED score in patients with AF [24]. However, this score does not consider variables related to off‐label dosing. Therefore, the HAS‐B“O”ED score was appropriate and novel for patients who underwent AF ablation and took DOACs.

### 4.5 Discontinuation of DOACs After AF Ablation

In RYOUMA registry, the interruption of DOAC varied according to CHADS_2_ scores [7], and a trend consistent with findings from database studies in Japan [25]. In this subanalysis, instead of using CHADS_2_ scores, patients were categorized into four groups: on‐label standard dose, on‐label reduced dose, off‐label underdose, and off‐label overdose. The incidence of stroke and thromboembolism was evaluated starting 90 days after CA, marking the end of blanking period. Additionally, the incidence of major bleeding was analyzed for each DOAC group in patients who continued DOAC therapy beyond 90 days. Low incidence rates limit definitive conclusions, but the results suggest that neither underdosing nor overdosing increased the risk of stroke or thromboembolism. Furthermore, there was no difference in the incidence of major bleeding among the groups during the perioperative period or within 30 days post‐CA.

### 4.6 Study Limitations

This study has several limitations. First, since the analyses were not conducted as part of a randomized controlled trial, caution must be exercised in deriving recommendations from the results. Second, the small number of patients enrolled in some subanalysis groups, and the insufficient number of thromboembolic events means that analyses of safety and efficacy outcomes may not have been adequate for each DOAC dose category, and the magnitude of differences may not have been large enough to identify in multivariate analyses adjusting for clinical risk factors. In addition, caution is needed due to differences in approved dosages and criteria for reducing DOAC doses in Japan compared to other regions. Finally, despite the prospective nature of this study, a significant limitation was seen in terms of the short follow‐up. In fact, the latest expert consensus statement [6] states that “in the intermediate‐risk patients (CHA_2_DS_2_‐VASc one in men and two in females) category, discontinuation of anticoagulation may be considered 12 months following CA in the absence of clinical symptoms or electrocardiographically documented AF recurrence.” Separate strategies may, therefore, need to be considered for anticoagulation therapy before and after 1 year following CA.

## 5 Conclusion

The proportion of patients receiving off‐label doses after CA for AF varied depending on the type of DOAC, ranging from 13.5% to 34.9%. Off‐label overdosing was infrequent, but significantly associated with a higher incidence of major bleeding events during the remote period after CA. Patients in the off‐label overdose group did not show any particular background characteristics, and the thromboembolic risk was, conversely, low. The most likely cause of off‐label overdosing was considered to be clinicians potentially overlooking dose criteria. Strict adherence to the dosage criteria is necessary.

## Ethics Statement

The ethical committee at each study site approved the study protocol before registry commencement, (University of Tsukuba Hospital: H28‐219). This study was conducted in accordance with the principles of the Declaration of Helsinki and the research protocol was approved by all applicable participating sites. All patients provided written informed consent before participating in the study.

## Conflicts of Interest

The authors declare no conflicts of interest.

## Data Availability

Deidentified participant data and the study protocol will be shared on a request basis for up to 36 months after publication of this article. Researchers who make such a request should include a methodologically sound proposal on how the data will be used; the proposal may be reviewed by the responsible personnel at Daiichi Sankyo, Co. Ltd., and the data requestors will need to sign a data access agreement. Proposals should be directed to akihiko-ind@umin.ac.jp.
